# Dysentery, a Clinical Study

**Published:** 1919

**Authors:** 


**Dysentery, a Clinical Study.** By Lieut.-Colonel John Cowan and Capt. Hugh Millier, R. A. M. C. *Journal Royal Army Medical Corps*, September, 1918.

The authors record a series of cases of dysentery drafted from the Western and Eastern theatres of war; all were very different in character and no exact statistics could be drawn up. Out of 600 cases, however, the entameba were detected in 58, a dysentery bacillus in 132, and lamblia in 50. Owing to the extensive movement of troops in this war, diseases indigenous to one place have been imported to another; thus, cases of dysentery have been recorded among men who had never left England. The persistent latency of protozoal infections is, on the other hand, responsible for a group of cases where infections acquired in another country show evidence of their existence when the carrier has reached some other part of the globe. In consequence, successful treatment of dysentery can only be ensured by an accurate diagnosis of the cause of each individual case.

*Amebic Dysentery.* A very large proportion of the population where amebic infections are endemic are carriers. Evidence of this is given by a number of observers, such as Strong, Musgrave, Craig, O'Connor, etc. The incidence of the infection is considerable, too, among men who are convalescent either from an attack of dysentery or enterica. Pure amebic dysentry, i. e., amebiasis unassociated with bacterial infection, is extremely rare, if at all existent. In the majority of cases the dysenteric symptoms subside under general treatment before the entameba are detected. A number of cases have double infection : both entameba and the dysentery bacillus are found in the stools, but, in amebiasis associated with dysenteric symptoms, the majority of patients show no dysentery bacilli, although other bacteria capable of producing dysenteric symptoms in a bowel already invaded by entameba are commonly present. An acurate diagnosis of amebic dysentery can only be made by microscopic examination of the stools, and the only real test is the discovery of the E. histolytica. In cases where microscopic examination is impossible, special attention should be paid to other data. The probable source of the infection and the nature of the dysentery there prevalent are particularly important. Recurring and chronic dysentery, as well as dysentery which fails to react to general treatment, or is associated with enlargement of the liver, hepatitis and perihepatitis, are generally amebic in origin.

The treatment consists in the administration of ipecacuanha or emetine in sufficient doses over a sufficient length of time.

*Bacillary Dysentery.* In the typical acute case the onset and the symptoms are those of an acute bacterial infection in which gastro-intestinal troubles prevail. In another group the symptoms can hardly be separated from those of the amebic type. The general features of the illness, epidemic in character, acute onset and severe toxemia, are the most characteristic symptoms of bacillary dysentery, but the only sure test is the recognition of the specific cause in the stools. In 58.8 o/o of the cases with blood and mucus in the stools, examined microscopically and bacteriologically, no dysentery bacilli or entameba were detected and the cause remained undetermined. In some cases, blood, mucus and pus persist after dysentery bacilli have ceased to be recovered; in others the bacilli persist after blood, mucus and pus have gone and the stools are normal.

